# Paediatric Calcaneal Osteochondroma: A Case Report and a Literature Review

**DOI:** 10.3390/diseases12080167

**Published:** 2024-07-25

**Authors:** Valeria Calogero, Michela Florio, Silvia Careri, Angelo Gabriele Aulisa, Francesco Falciglia, Marco Giordano

**Affiliations:** 1 U.O.C Traumatology, Bambino Gesù Children’s Hospital, IRCCS, 00165 Rome, Italy; 2 Department of Human Sciences, Society and Health, University of Cassino and Southern Lazio, 03043 Cassino, Italy

**Keywords:** calcaneal osteochondroma, exostosis, multiple osteochondromas, heel pain

## Abstract

Background: Heel pain in children is a common condition. The aetiology can be ascribed to fractures, osteochondrosis, tendinitis, calcaneal-navicular or talo-calcaneal coalition, osteomyelitis, rheumatic diseases, anatomic variants, malignant tumours (osteosarcoma, Ewing’s sarcoma), and benign lesions (bone cyst, aneurismal bone cyst, osteoid osteoma, or exostosis). In particular, this manuscript focuses on a case of calcaneal exostosis in the paediatric age, aiming to highlight its rarity. Osteochondromas are benign tumours of the surface of the bone and the overlying cartilage. They grow until skeletal maturity and can cause stiffness, pain, cosmetic alterations, tendinitis, and neuro-vascular compression. The calcaneus is an extremely rare site for these tumours. Only two case reports of paediatric exostosis of the calcaneus bone are available. Methods: We describe a case of a girl of 16 years of age, affected by multiple cartilaginous exostosis, who presented with a painful mass on the inferior margin of the foot in the calcaneal region, which was diagnosed as an exostosis. The neoformation was excised, and the girl underwent clinical follow-up. Results: The patient was promptly discharged in good condition, and on the 25th postoperative day, she was completely pain-free and allowed weight bearing. Conclusions: In the case of heel pain resistant to conservative treatment, the presence of an osteochondroma should be considered after excluding more common causes. If symptomatic, calcaneal osteochondromas could require surgical excision.

## 1. Introduction

Heel pain in children is common and can be attributed to different conditions. Therefore, it is important to make a correct differential diagnosis, taking into account the most frequent pathologies.

Concerning traumatic pathology, special attention should be paid to occult fractures in very active children and to stress fractures in those who participate in high-impact activities such as running and jumping. Another cause of heel pain is represented by Sever–Blanke disease, an osteochondrosis which results from repetitive microtraumas to the secondary ossification centre of the calcaneus due to the traction of the Achilles tendon insertion, with the development of a painful traction apophysitis [1]. In the case of the medial arch and heel pain in children who participate in running or jumping sports, plantar fasciitis can be seen, and it is often associated with Sever’s apophysitis in the young athlete. If the pain is referred to appear mainly on the anterior and lateral aspects of the ankle, we should consider the existence of a calcaneal-navicular or talo-calcaneal coalition. In the presence of risk factors such as immunosuppressive conditions, diabetes mellitus, history of puncture by insects, and systemic inflammatory or infective conditions, we should exclude the possibility of osteomyelitis. The calcaneus is involved in 4–11% of the cases of osteomyelitis in children, and the responsible pathogens are usually Staphylococcus aureus, Streptococcus, Escherichia coli, Klebsiella, and Candida albicans [2]. Juvenile rheumatoid arthritis and psoriatic arthritis of the foot are relatively uncommon [3]. There could also be anatomical variants of the calcaneus that clinically present as circumscribed, palpable swellings. Malignant neoformations of the bone in children are rare (3–6% of all paediatric bone tumours), and they are represented by osteosarcoma or Ewing’s sarcoma of the calcaneum. Regarding benign neoformations, it is important to evaluate the presence of simple bone cysts, aneurismal bone cysts, osteoid osteomas, and exostoses.

Regarding osteochondromas (or exostoses), they are developmental-age benign neoformations of the bone surface and overlying cartilage, accounting for about 36–41% of all benign bone tumours [4]. Osteochondromas in the ankle and foot are uncommon, and the calcaneus bone is an extremely rare site. However, several regions of this bone may be involved. Osteochondromas typically grow during childhood until skeletal maturity and can have a sessile or pedunculated morphology. In 60% of such cases, these neoformations are observed in patients younger than 30 years of age.

Some patients are diagnosed with a systemic condition called multiple exostoses, also known as Bessel Hagen disease, multiple osteochondromas, multiple cartilaginous exostoses, hereditary deforming chondrodysplasia, or osteochondromatosis, whose prevalence is 1:50,000 [5], with a male-to-female ratio of 1.5:1 having an autosomal dominant inheritance. This pathology is due to certain mutations in the genes EXT1 (8q24.11) and EXT2 (11p11.2), respectively, resulting in multiple exostoses of type 1 and type 2. These genes code for a protein called exostosin, involved in heparin sulphate proteoglycan synthesis, which has a role in the proliferation of the cartilaginous growth plate. About 15% of people with hereditary multiple exostoses have no mutation in either the *EXT1* gene or the *EXT2* gene. Multiple exostoses are also seen in a condition called metachondromatosis and in the Langer–Giedion syndrome. Exostosis-like lesions also occur in fibrodysplasia ossificans progressiva, occipital horn syndrome, and hereditary hypophosphatemia. The existence of an anatomical variant of the distal anteromedial humeral region should be considered, which can be confused with an osteochondroma and is present in 1% of Caucasian patients.

Multiple osteochondromas disease is supposed to be present when at least two osteochondromas are detected in the same patient. It is important to highlight that to be classified as exostoses, the neoformations have to maintain continuity with the bone cortex, the perichondrium, and the medullary canal. Trabeculae of the hyaline cartilage make up the cap structure, with chondrocytes arranged in lines and surrounded by a perichondrium [6]. 

The average number of locations is 15–18 but may vary significantly, involving endochondral bones [7]. The bones developed through enchondral ossification, in particular long bones in their juxta-epiphyseal area, are most commonly affected: the distal femur (70%), the proximal tibia (71%), the humerus (50%), the ulna and radius (30%), and the proximal fibula (27%). Less commonly, the condition affects the hands, the thorax, the scapulae, the spine, and the pelvis [8]. Osteochondromas in the ankle and foot are not common. Only 10% of osteochondromas affect the bones of the hands and the feet [9].

Signs and symptoms may vary from slight cosmetic alterations to significant compression of anatomic structures with pain and organ disorders, having an impact on the psychological, physical, and social aspects of children’s quality of life.

The severity of symptoms associated with exostoses seems to be greater in type 1. They can cause bony deformities; growth disturbances when involving the physis; leg length discrepancy [10]; local inflammation with the formation of bursae; arthritis (14%); pain; compression on adjacent structures such as tendons, nerves (22.6%), blood vessels (11.3%), and the spinal cord (0.6%) [11]; articular impingement with stiffness and reduction in the articular range of motion [12]; and cosmetic alterations. Rarer complications reported in the literature are represented by dysphagia due to vertebral exostoses, haemothorax in the case of rib exostoses, gastrointestinal and urinary obstructions if they involve the abdomen and pelvis, and abnormalities in the vaginal canal leading to difficulties in delivery [13]. Considering the symptoms and the presence of deformities and/or functional limitations, multiple exostoses disease can be classified through the classification of Mordenti et al. [14].

Multiple exostoses disease is considered a chronic condition, having a relevant impact on the psyche and quality of life. Children could present with a real disability, with problems at school, self-enclosure, difficulties in social relations, and avoidance of sporting activities [15].

It is worth mentioning that exostoses can also appear in Gardner syndrome, a severe form of autosomal dominant familial adenomatous polyposis with multiple adenomas of the colon and rectum [16].

The natural course of exostoses is for them to finish growing when the epiphyseal growth plates close. If the growth continues into adulthood, a suspicion of malignant transformation is suggested. The thickness of the cartilaginous cap is suggestive of malignant transformation if greater than 2–3 cm. The transformation is estimated to occur in less than 1–2% of patients with solitary osteochondroma and 5–25% of patients with multiple hereditary exostoses [17]; usually, the diagnosis is of low-grade chondrosarcoma or secondary osteosarcoma. The most affected sites are the appendicular skeleton (87%), particularly the pelvis, then the scapula, the proximal femur, and the thorax in terms of vertebrae and ribs [18].

Concerning instrumental diagnosis, plain radiographs of the interested site are widely used and allow us to identify suspected osteochondromas and bony deformities. Also, ultrasound can play a role in the detection and be used to measure the hyaline cartilage cap thickness in some selected cases. However, ultrasound is not easy to perform in obese patients, and its variability depends on the operator’s experience. CT examination can be useful in the most complex cases for surgical planning of neoformation removal and the correction of deformities, especially if thin sections are performed, because the bi-dimensional and three-dimensional reconstructions give us an idea of the appearance of the neoformation and the structures around it. MRI can help us to accurately study the thickness of the cartilage cap, the neighbouring soft tissues, and the relationship with the neuro-vascular bundles. For early detection of malignant transformation in patients with multiple exostoses disease, a whole-body MRI scan can be useful. Also, bone scintigraphy and positron emission tomography with 18-fludeoxyglucose can play a role [19]. Molecular genetic testing is necessary to confirm the postulated diagnosis [20].

With this case report, we describe a rare case of heel pain related to the presence of a calcaneus osteochondroma in a patient affected by multiple exostoses disease.

## 2. Clinical Case

A girl suffering from multiple cartilaginous exostoses presented to our institution in 2011 at the age of 5. The patient underwent multiple surgeries due to the underlying pathology. We excised osteochondromas from different sites: two from the distal right ulna were removed at the age of 5 years, another one from the distal metaphysis of the left tibia at the age of 6 years, two exostoses from the postero-lateral surface of the distal metaphysis of the left femur at the age of 7 years, one from the proximal metaphysis of the right radius and one from the ulna at the age of 7 years and 8 months, two osteochondromas from the proximal epiphysis of the right radius and one from the proximal third of the left humerus at the age of 9 years, another osteochondroma from the distal metaphysis of the left femur and one from the proximal metaphysis of the left tibia at the age of 10 years, an osteochondroma from the distal third of the right tibia at the age of 11 years, and one from the right tibia at the age of 12 years. 

In September 2023, at the age of 16 years and 10 months, she presented with pain on the inferior aspect of the foot in the calcaneal region, described as having been present for about a year. During the last few months, the girl had perceived the presence of a palpable mass. She did not experience any trauma. She could not play sports and had a functional limitation because of pain and discomfort. On the previous days, the patient had medical treatment with non-steroidal anti-inflammatory drugs without any benefit; she avoided sports and walking with her weight resting on her heel, but the soreness was relevant. The patient had no symptoms and no suspicious signs of recent infection or systemic inflammation, and the pain was well located in the heel area. 

**Figure 1:**
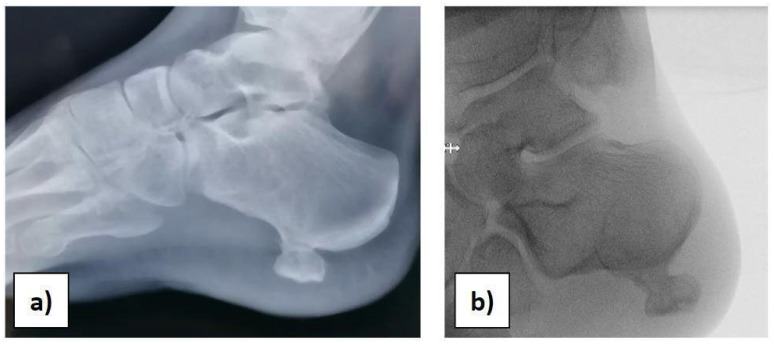
(**a**) Preoperative X-ray. (**b**) Intraoperative X-ray before starting the surgery.

At the orthopaedic clinical examination, a stiff, immobile, and painful mass measuring approximately 2 × 1.5 cm was palpated in the heel region. The range of motion of the ankle was complete. The girl was subjected to an X-ray examination of the foot in anterior–posterior, later-lateral, and oblique projections, which revealed a 2 × 1, 5 × 1 cm pedunculated bone lesion involving the load-bearing part of the heel, arising from the posterolateral aspect of the calcaneus, with no fracture signs (Figure 1a,b). 

**Figure 2:**
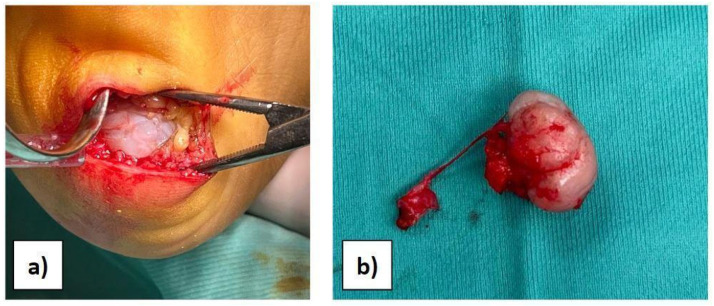
(**a**) View of the bony mass through surgical access. (**b**) The excised mass.

Considering the results of imaging studies, the patient’s persistent and progressive heel pain, and the diagnosis of multiple exostoses disease, surgery was proposed and accepted by the family. With the patient supine and under general anaesthesia, after intravenous administration of antibiotic, antisepsis and sterile field preparation were performed; then, a transverse incision of approximately 4 cm was made on the lateral plantar side of the hindfoot. A full-thickness approach was performed to avoid damage to the neurovascular tissues, reducing the risk of skin necrosis. Subsequently, the cartilaginous cap was observed (Figure 2a), and the mass was isolated. Under magnification, the bone mass was excised through an osteotomy at the base of the pedicle (Figure 2b). Complete excision was verified by intraoperative X-ray. The surgical site was then irrigated, the tissues were closed, and the skin was sutured with resorbable suture thread in order to avoid aesthetic problems.

The excised tissue was then examined by the pathologist, who confirmed the diagnosis of osteochondroma. The patient was discharged the day after intervention with instructions to use two crutches and avoid weight-bearing on the operated foot; then, a postoperative visit was scheduled. At the physical examination on the 25th postoperative day, the patient was pain-free; the wound was well closed with good scar formation; and she was allowed to bear weight. After 19 months from surgery, she stated that she was satisfied with the intervention performed, claiming to have resumed all physical activities without any pain and to have a good quality of life without restrictions.

## 3. Discussion

**Figure 3:**
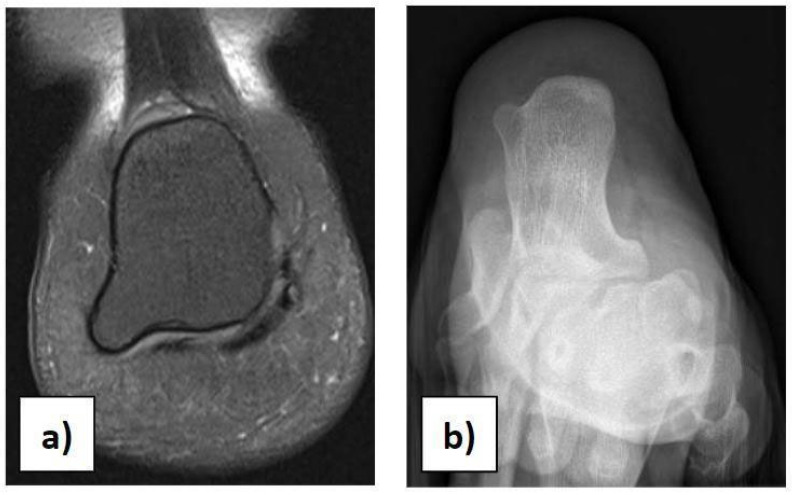
(**a**) Anatomical variation in the calcaneus on MRI. (**b**) Anatomical variation in the calcaneus on X-ray.

In the case of children with heel pain, it is important to perform a proper clinical assessment to make a suitable differential diagnosis, excluding traumas, occult or stress fractures, osteochondrosis, calcaneo-navicular or talo-calcaneal coalition, anatomical variations, rheumatologic conditions, infections, benign neoformations, and malignant neoformations. Anatomical variations in the calcaneus should be considered in the case of palpation of masses during physical examination [21]. We had a case of a 12-year-old girl with an incidental X-ray and MRI finding of a painless 7 × 8 mm bilateral symmetric bony mass on the infero-lateral aspect of both calcanei, without a cartilaginous cap (Figure 3a,b). She could play all sports and wear footwear without any problems and did not need any orthopaedic treatment.

In the absence of more common causes, especially in children with a diagnosis of multiple exostoses disease, we should consider the presence of a calcaneal osteochondroma.

To our knowledge, there are only a few reports of adult osteochondromas in the literature [22,23,24,25,26,27,28,29,30,31] and only two cases of paediatric osteochondromas of the calcaneal bone. Nasir et al. [32], in 2020, presented a 15-year-old male patient with a sessile bone growth of 35 mm × 26 mm on the inferolateral aspect of the right calcaneus, which was excised with no recurrence at 1-year follow-up and with a histopathological benign diagnosis 

Mostafa et al. [33], in 2021, presented a 10-year-old child with an osteochondroma of the lateral wall of the calcaneus with a confirmed diagnosis of osteochondroma after removal and without recurrence at 1-year follow-up.

The sinus tarsi could also be involved. Andreacchio et al. [34] described a case of an 8-year-old patient who presented with recurrent ankle sprains and a reduction in the range of motion of the subtalar joint and who underwent surgical excision with good clinical outcomes. Sung Hun Won et al. [35] reported a 15-year-old patient who showed pain and medial plantar forefoot paraesthesia, with a normal range of motion of the subtalar joint, and who underwent surgical excision and nerve release for a sinus tarsi exostosis and showed no recurrence at a 2-year follow-up. 

We suggest a clinical follow-up after excision surgery every 12 months until skeletal maturity. In the event of recurrence of pain or swelling, radiography is considered the standard examination, followed by MRI, in consideration of the possibility of recurrence and malignant transformation.

The case presented aims to highlight the rarity of calcaneal exostosis in paediatric-age individuals. Due to the rarity of the lesion, the diagnostic process may be delayed. 

The symptomatology of calcaneal osteochondromas, compared to other sites, is represented by heel pain that may limit walking, swelling, callus formation, and a palpable mass. Clinical evaluation, genetic assessment, and imaging modalities allow us to orientate ourselves towards the correct diagnosis. Radiographs are the most used and are often diagnostic for osteochondromas, revealing many of the classical pathological characteristics, such as the orientation of the lesion that grows away from the physis while maintaining medullary continuity. Asymptomatic osteochondromas are commonly diagnosed incidentally on radiographs. MRI is useful in the measurement of cartilage thickness and the evaluation of the surrounding soft tissues. The CT scan helps us in the preoperative planning of complex cases. CT or MRI three-dimensional model printing is a valuable aid in complex surgical procedures and it is based on software-processed images that exactly replicate the morphology of the body segment, allowing an accurate study of the neighbouring structures and neurovascular bundles. US and scintigraphy can have complementary roles. Genetic testing is indispensable and allows us to orientate ourselves towards a correct framing. Histopathological examination of the excised mass is essential for certainty of diagnosis and for excluding malignant transformation [36].

The size and nature of the lesion, and the signs and symptoms, generally guide the optimal treatment. The treatment of asymptomatic exostoses of the foot is usually conservative, and a follow-up with radiographs [37] or MRI is recommended. Considering the weight-bearing nature of the foot, even small foot lesions may be symptomatic and require surgical excision. It is important to remember that any remaining cartilage cap may result in recurrence. Marginal resection and histological examination are the appropriate treatments, with a low recurrence rate of about 2% [38]. Possible complications related to the operation can involve the bone parts and the joints with iatrogenic fractures, synostosis, rotational deformity, osteochondroma pedicle fracture, stiffness of the surrounding joints, and reduction in the range of movement. In addition, the soft tissues can be involved in terms of bursitis, tenosynovitis, and tendon rupture, and the neuro-vascular bundles can be involved with neuropathy, vascular and nerve injuries, thrombosis, pseudoaneurysm, acute ischaemia, and phlebitis. Wirganowicz et al. reported a total complication rate of 12.5% for 285 excision surgeries [39].

It is important to keep in mind the existence of the possibility of malignant transformation. Suspicion must be raised in cases of growth after skeletal maturity, rapid growth, big lesions, and the appearance of pain in a previously stable lesion. An MRI cartilage cap thickness >2 cm in adults and >3 cm in children is interpreted as indicative of cancerous degeneration. This transformation is estimated to occur in less than 1–2% of cases of solitary osteochondroma and is represented by chondrosarcoma in most cases; less than 10% are osteosarcomas, fibrosarcomas, or spindle-cell sarcomas. The 10-year survival rates are 83% for grade I and 29% for grade III chondrosarcomas. In these tumours, the architecture of the cap is altered, appearing lobulated due to fibrous septa, and the tumour can encroach on surrounding tissues. In most cases, the surgical treatment of the lesion includes en bloc resection of the neoformations with free margins. If a complete resection is performed in regions such as the pelvis, reconstructive techniques should be considered [40].

In the case of children with multiple exostoses disease, there are some ongoing studies on the effectiveness of biological drugs. In particular, retinoic acid receptor agonists (RARγ) have been evaluated because of their involvement in cartilage growth. Further studies are needed to establish the role of pharmacological target therapy [41].

## 4. Conclusions

In cases of heel pain resistant to conservative treatment, if common conditions such as traumas, osteochondrosis such as Sever–Blanke disease, tendinitis, and infections are excluded, an osteochondroma should be considered. Anatomical variations, rheumatologic diseases, and malignant tumours represent rarer conditions.

Exostoses are benign neoformations that may present as solitary or in the context of multiple exostoses disease. Osteochondroma of the calcaneus is uncommon but is a potential cause of debilitating heel pain. Only two cases of proper calcaneal bone exostosis in the paediatric population are available in the literature. Physicians should assess the clinical signs and symptoms, empathise with the child’s needs, and analyse the results of instrumental examinations to make a correct diagnosis and ensure effective treatment and a good quality of life. Surgical excision should be considered in cases of heel pain, walking limitation, and close relationship with neurovascular bundles or tendons. A marginal resection may improve symptoms and reduce heel pain.

## Data Availability

Datasets generated and/or analysed during the current study are available from the corresponding author upon reasonable request.
